# Histone Modifying Enzymes as Targets for Therapeutic Intervention in Oesophageal Adenocarcinoma

**DOI:** 10.3390/cancers13164084

**Published:** 2021-08-13

**Authors:** Oliver J. Pickering, Stella P. Breininger, Timothy J. Underwood, Zoë S. Walters

**Affiliations:** School of Cancer Sciences, Faculty of Medicine, University of Southampton, Southampton SO17 1BJ, UK; o.j.pickering@soton.ac.uk (O.J.P.); s.p.breininger@soton.ac.uk (S.P.B.); T.J.Underwood@soton.ac.uk (T.J.U.)

**Keywords:** oesophageal cancer, epigenetics, biomarker, histone modifying enzymes, EZH2, HDAC

## Abstract

**Simple Summary:**

Despite advances in both the surgical and medical management of oesophageal adenocarcinoma (OAC), overall prognosis remains poor, with less than 1 in 5 patients surviving more than 5 years from diagnosis. Patients suitable for treatment with curative intent receive pre-operative chemotherapy, but few (<20%) gain a clinically meaningful response. Epigenetic targets have been identified as key drivers in OAC. With therapies targeting histone-modifying enzymes (HME) already in clinical use in several cancer types, they may be a potential novel therapeutic in OAC. This review outlines the current literature surrounding the roles of HMEs in OAC tumorignesis and therapeutic efficacy.

**Abstract:**

Oesophageal adenocarcinoma (OAC) has a dismal prognosis, where curable disease occurs in less than 40% of patients, and many of those with incurable disease survive for less than a year from diagnosis. Despite the widespread use of systematic chemotherapy in OAC treatment, many patients receive no benefit. New treatments are urgently needed for OAC patients. There is an emerging interest in epigenetic regulators in cancer pathogenesis, which are now translating into novel cancer therapeutic strategies. Histone-modifying enzymes (HMEs) are key epigenetic regulators responsible for dynamic covalent histone modifications that play roles in both normal and dysregulated cellular processes including tumorigenesis. Several HME inhibitors are in clinical use for haematological malignancies and sarcomas, with numerous on-going clinical trials for their use in solid tumours. This review discusses the current literature surrounding HMEs in OAC pathogenesis and their potential use in targeted therapies for this disease.

## 1. Introduction

### 1.1. Oesophageal Adenocarcinoma

Oesophageal cancer is the ninth most common malignancy worldwide, with an estimated global cancer burden of 572,034 cases and 508,585 deaths in 2018 [1]. It has a dismal prognosis with less than 20% of patients surviving 5 years after diagnosis [2,3,4]. Oesophageal cancer is comprised of two major, histologically distinct subtypes: oesophageal squamous cell carcinoma (OSCC) and adenocarcinoma (OAC). OSCC accounts for 70% of global oesophageal cancer cases but has seen a substantial fall in incidence in recent years, owing to improved living conditions and a decrease in the prevalence of tobacco smoking [5,6,7]. In contrast, OAC has seen a sustained rise in incidence in Western populations since the late 1980s, with incidence rates surpassing that of OSCC in the UK in 1985 [8,9]. OAC is highly aggressive and is usually diagnosed at a late stage, mainly due to the delayed nature of symptomatic presentation, with approximately 40% of patients presenting with stage 4 disease [10]. Despite advancements in both the medical and surgical management of OAC, less than 40% of patients are suitable for treatment with curative intent [1,10].

Gastro-oesophageal reflux is the most important risk factor for OAC with obesity, older age and smoking also significant factors [7]. OAC is believed to develop through a metaplasia-dysplasia-neoplasia sequence, with chronic inflammation secondary to reflux resulting in Barrett’s oesophagus, a precancerous condition in which the normal oesophageal squamous epithelium is replaced by metaplastic columnar epithelium, later progressing to adenocarcinoma [11]. Numerous molecular changes occur during the conversion of normal epithelium to OAC, with widespread genetic and epigenetic changes underlying this multi-step process [11].

Management of OAC is dependent on patient characteristics, as well as tumour stage and position. Surgery is the mainstay of curative treatment in combination with neoadjuvant chemotherapy or chemoradiotherapy. Despite its widespread use, less than 20% of OAC patients show a clinically significant response to chemotherapy, with the remaining patients undergoing neoadjuvant treatment with its many associated toxic side effects without benefit [12]. OAC remains a cancer of unmet need and a current research priority. An emerging area of interest is the role of epigenetic regulators in OAC pathogenesis, and these may provide potential novel avenues for future therapy, which we review here.

### 1.2. Epigenetics Overview

The term ‘epigenetics’ was first proposed in 1942 to describe changes in cellular phenotype without changes in genotype due to complex and poorly understood mechanisms [13]. The predominant epigenetic mechanisms regulating gene expression can be separated into four distinct categories: (1). DNA methylation; (2). covalent histone modifications; (3). non-covalent modifications including histone variants and nucleosome positioning; and (4). non-coding RNAs including microRNAs [14]. The interaction of these mechanisms, which are occurring simultaneously and are collectively referred to as the epigenome, influences the folded structure of the chromatin, regulating the availability of regions of DNA to act as a template for replication, recombination and transcription [15]. Epigenetic mechanisms allow the regulation of gene expression patterns through adapting chromatin, whilst leaving the underlying DNA sequence unchanged. These mechanisms, together with other mechanisms of transcriptional regulation, are considered key to both normal cellular function and abnormal disease processes [15]. Technological advances have enabled in-depth analysis of the epigenome, facilitating a rapid expansion of our understanding in this field, with epigenetic regulation in cancer of increasing interest [16,17]. Improvements in our understanding of epigenetic mechanisms at the centre of disease pathogenesis is facilitating research into developing biomarkers for patient stratification and epigenetic-based therapies [16].

The role of epigenetics in the development of Barrett’s oesophagus and progression to OAC has predominately focused on DNA methylation [11,18,19]. Genome-wide analysis has revealed that global hypomethylation is the dominant change during the formation of Barrett’s metaplasia, with selective promoter site hypermethylation at sites such as *P16* and *Secreted frizzled related protein (SFRP)* tumour suppressor genes seen during the later stages of OAC pathogenesis [11]. Despite promising results for therapies targeting histone-modifying enzymes (HME) being seen in clinical trials, particularly in haematological malignancies where there are currently four US Food and Drug Administration (FDA) HME inhibitors, they have received little attention in OAC research and have not yet been reviewed [20,21,22,23,24,25,26]. This review focuses specifically on the role of HMEs in OAC and discusses their potential as novel therapies for this disease.

### 1.3. Histone Modifications

Histones are vulnerable to a variety of post-translational modifications through the enzymatic alteration of their amino (N)-terminal tails, which are accessible domains that protrude out of nucleosomes, allowing contact with adjacent nucleosomes. HMEs are responsible for covalent histone modifications including methylation, acetylation, phosphorylation and ubiquitination, playing fundamental roles in both normal and dysregulated biological processes [27,28]. These dynamic modifications do not occur in isolation, meaning transcription activation and repression are controlled by the interplay of numerous HMEs, together with other epigenetic mechanisms, to influence chromatin structure and therefore, gene expression [29,30]. Given the dysregulated activity of HMEs in cancer and their numerous histone and non-histone targets, HMEs are in the focus of cancer researchers [31,32]. To date, the limited work on HMEs in OAC has identified roles for histone methylation and acetylation in tumorigenesis, with few studies reporting on HME-targeted therapies for OAC (Table 1).

## 2. Histone-Modifying Enzymes in OAC

### 2.1. Histone Deacetylases in OAC Pathogenesis

During histone acetylation, an acetyl group is transferred from the co-factor acetyl CoA to the amino acids of histones. This occurs at lysine residues and is regulated by the opposing actions of histone acetyltransferases (HAT) and histone deacetylases (HDAC) [27]. Acetylation of the histone tails neutralises the lysine’s positive charge, which has the potential to weaken the interaction between the tail and the negatively charged nucleosomal DNA [30]. This results in an opening of the chromatin into the more transcriptionally active euchromatin state.

In order to examine the role of HDAC1 in OSCC and OAC, Miyashita et al. (2014) surgically induced chronic duodenal reflux oesophagitis in rats, allowing changes in histone acetylation to be investigated during OAC development [38,39,40,41]. A proportion of rats were sacrificed sequentially at 10-week intervals post-surgery and their resected oesophagi specimens were examined histologically. By 50 weeks post-surgery, all the sacrificed rats (*n* = 15) had areas of Barrett’s metaplasia, with 40% (6/15) also having histological evidence of OAC and 13% (2/15) having OSCC. When examining expression of HDAC1 and metastasis-associated gene 1 (MTA1), a component of the HDAC containing a nucleosome remodelling ‘NuRD complex’, there was no expression in either protein in normal oesophageal squamous epithelium. Contrarily, HDAC1 and MTA1 were expressed in all stages of squamous and adeno- carcinogenesis. These findings are consistent with a variety of cancer cell lines including breast, ovarian, gastric and pancreatic cells, where overexpression of MTA1 and NuRD complexes have been detected [42]. In both non-small cell lung cancer cells and human hepatoma cells, NuRD complexes have been shown to exert deacetylation activity against the tumour suppressor *TP53*, which results in an increase in its degradation by MDM2 and a reduction in TP53-mediated cell growth arrest and apoptosis [43,44]. Miyashita et al. (2014) conclude that their results suggest HDAC1/MTA1 complexes are involved in the pathogenesis of oesophageal cancer and may provide future targets for OAC treatments. However, given that *TP53* loss of function mutations are seen in over 70% of OACs [45], it is unclear whether increased NuRD complex expression and its impact on *TP53* mediated cell growth arrest is only relevant in *TP53* wild-type OAC. Overexpression of MDM2, responsible for degradation of *TP53* after deacetylation by NuRD complexes, has been shown to be mutually exclusive with *TP53* mutations and so a similar relationship may exist with NuRD complexes and *TP53*, although this has not yet been demonstrated [45].

Langer et al. (2010) analysed HDAC1 and HDAC2 expression by immunohistochemistry (IHC) in 179 OAC patients, out of which 131 resection specimens were obtained from neoadjuvant treatment naïve OAC patients (oesophagectomy only), and in 48 patients with locally advanced OAC treated with conventional neoadjuvant chemotherapy followed by surgery [46]. Moderate or high HDAC1 expression was seen in 46% (80/179) of tumours. In contrast, 70% (106/179) of tumours had moderate or high HDAC2 expression. Higher HDAC2 expression correlated with lymphatic tumour spread (*p* = 0.046) and lower tumour differentiation grade (*p* = 0.002). Associations between HDAC1 or HDAC2 expression with neoadjuvant chemotherapy were evaluated by comparing HDAC1 or HDAC2 expression in pre-therapeutic tumour biopsies against histopathological tumour regression after treatment. In the available 37 specimens, high HDAC2 expression showed a non-significant trend with an improved response to chemotherapy, which may be limited by the studies’ small sample size. No significant association was seen between HDAC1 or HDAC2 expression and overall survival. The authors suggest that high HDAC expression may represent a marker for aggressive tumour behaviour in OAC but acknowledge the limited sample size of the study and the non-statistically significant findings. Our unpublished analysis of TCGA Research Network data on OAC (*n* = 80) revealed that high expression of *HDAC1* (HR 2.22 (1.15–4.29), *p* = 0.015) and *HDAC2* (HR 2.38 (1.25–4.54), *p* = 0.006) is associated with a worse prognosis in bulk transcriptome RNA datasets [47]. This is further supported by observations in prostate cancer, where high expression of *HDAC1* is associated with lower tumour cell differentiation and increased cell proliferation [48]. Similarly, in breast cancer, higher *HDAC1* expression correlated with more aggressive tumours [49]. In contrast, a correlation between advanced tumour stages and decreased HDAC1 expression has been observed in OSCC [50].

Genetic aberrations in the known proto-oncogene *Myc* has been shown to cause upregulation in *HDAC* expression in a number of malignancies, including colon cancer and neuroblastoma [51,52,53]. Whilst attempts to therapeutically target *Myc* throughout cancer have been largely unsuccessful, HME therapies have shown promise [54,55]. Both HDAC inhibitors (HDACi) and inhibitors of Bromodomain and Extra-Terminal motif (BET) protein, which are required for the recognition of acetylated lysine residues in histones, have been shown to have anti-tumour activities against numerous *Myc*-amplified cancers including neuroblastomas, acute myeloid leukemia, myeloma and prostate cancer [55,56,57,58]. Whilst a similar relationship between *Myc* and *HDAC* expression has not been reported in OAC, *c-Myc* is found upregulated in over 80% of OAC and so has the potential of being targeted through HME therapies [59,60].

### 2.2. HDAC Inhibitors for the Treatment of OAC

Much of the current research on OAC HME therapies has been reported on HDACi. There are currently over 20 HDACi in clinical trials for the treatment of cancer. The most extensively studied are non-selective HDACi [20]. Vorinostat (SAHA) was the first to be approved by the FDA in 2006 for the treatment of primary cutaneous T-cell lymphoma (CTCL) [23], which was followed by the FDA approval of belinostat and panobinostat for use in peripheral T-cell lymphoma (PTCL) [24] and multiple myeloma [25] respectively. There are also numerous selective HDACi in clinical trials, with most displaying selectivity for class I and class II HDACs. Romidepsin, an HDAC1 and HDAC2 inhibitor, was the first to be approved by the FDA for treatment of CTCL and PTCL [26] and there are promising results seen in pre-clinical trials for inhibitors of HDAC3 [61], 4 [62], 5 [63] and 8 [64] in various cancer types.

Lohse et al. (2018) evaluated the treatment response towards a panel of 215 FDA-approved anti-cancer drugs and 163 epigenetic compounds using established OAC lines (OE33, OE19, FLO1 and SKGT2) and two patient-derived OAC cell lines (EAC42 and EAC47) [33]. When focusing on epigenetic therapies, 10 of the 163 compounds tested displayed significant antitumour activity, classified as cell killing greater than 3 standard deviations above negative control, in all 6 included cell lines. With the exception of a polo-like kinase 1 (PLK1) inhibitor, the 9 remaining compounds were HDACi, providing the basis for further study focusing on HDACi-based treatments.

In order to allow direct chemo-sensitivity testing and screening of novel drugs including HDACi on OAC cells within the complex ecosystem of the tumour microenvironment, Saunders et al. (2017) derived 3D- tumour growth assays (3D-TGA) from pre-treatment tumour biopsy specimens [34]. Tumour growth assays with or without supporting mesenchymal cells showed sensitivity to achievable serum concentrations of panobinostat (HDACi) monotherapy, but the presence of mesenchymal cells led to a 1-fold increase in required panobinostat concentrations. Standard of care triplet chemotherapy with epiribucin, cisplatin and fluorouracil (ECF) were given in combination with panobinostat, resulting in increased treatment efficacy, with only 2/12 ECF-resistant samples remaining resistant after combination therapy.

Ahrens et al. (2015) assessed the effect of HDACi and/or DNA methyltransferase inhibitors (DNMTi) in OSCC and OAC [35]. In the two included OAC cell lines (OE33 and SKGT4), HDAC activity was increased compared to a non-neoplastic epithelial cell line (Het-1A), which reflected expression patterns observed in ten case-matched tissue specimens of normal oesophageal epithelium and OAC from neoadjuvant treatment naïve patients. OE33 cell lines were subject to treatment with one of three HDACi, the non-selective inhibitor vorinostat or the selective inhibitors MS-275 and FK228, or one of two DNMTi, azacytidine (AZA) or decitabine (DAC). After 72 h of treatment, both MS-275 and FK228 effectively reduced OAC cell viability, but no significant reduction was seen with vorinostat. AZA showed a significant effect on OE33 at the highest dose of 100μM only, whereas DAC treatment was infective at all doses (up to 100 μM).

When combining MS-275 and AZA, a marked reduction in OE33 cell viability was observed which was greater than either treatment alone. Interestingly, combination treatment did not affect control HET-1a cells, despite having greater HDAC expression, indicating that these agents selectively targeted OAC cell lines. Ahrens et al. (2015) demonstrated that combination treatment exerted anti-tumour effects by inducing DNA damage, cell viability loss and apoptosis, and by decreasing cell migration. Whilst further study is needed in this area, the synergistic effects of HDAC and DNMT inhibition in OAC looks promising, with identification of candidate genes providing a platform for detailed functional pathway analysis and investigation into biomarkers of treatment response.

The promising results seen with HDACi in OAC in vitro studies have been further supported by Feingold et al. (2018) who evaluated the role of Thioredoxin Interacting Protein (*TXNIP*), a hypothesised tumour suppressor in OAC, that has previously been shown to be regulated by histone acetylation [36,65]. Using several OAC cell lines, baseline *TXNIP* gene expression was analysed using qRT-PCR, immunoblot, and immunofluorescence techniques and was found to be significantly lower in OAC cells compared to normal oesophageal tissue. *TXNIP* overexpression through lentiviral cDNA expression in murine xenograft OAC models led to increased sensitivity of OAC to cisplatin mediated apoptosis in these models. To further explore this, OAC cells were treated with a combination of cisplatin and the HDACi entinostat, which led to an upregulation in the expression of *TXNIP*, confirmed by an increase in the H3K27Ac activation mark at the *TXNIP* promoter. The combination therapy acted synergistically to increase DNA damage and apoptosis of the OAC cells via the mitochondrial apoptotic pathway, providing evidence of the possible roles of HME inhibitors (HMEi) as combination therapies in OAC.

Kofonikolas et al. (2019) (conference abstract only) investigated the effects of BET or HDACi on OAC cell lines (SKGT4, JHesoAD, OACP4C, OACM5.1, FLO1, OE19 and ESO51) including a cell line with amplifications of the known oncogene *Myc* [37]. *Myc*-amplified OAC cells showed increased sensitivity to BET inhibition at similar concentrations used in melanoma and lymphoma cell lines, where clinical activity has been observed [37]. The OAC cell lines were less sensitive to HDACi, requiring much higher concentrations of vorinostat to reduce cell proliferation.

Despite the promising early results seen with HDACi in vitro they have been slow to progress to pre-clinical studies in OAC, which is likely due to numerous factors. We speculate that the overall sparsity of research focused on HME in OAC has limited progress with novel therapies in this area. As our knowledgebase improves, we suspect we will see an increase in pre-clinical and clinical research with epigenetic therapies. Secondly, whilst 3D OAC models exist, they are not easily reproducible and so 2D cell lines remain the predominant form of in vitro models. Without an attempt to mimic the impact of the TME, there is an increased risk of in vitro results not transferring to positive in vivo results. Finally, the lack of specific biomarkers for patient selection for HDACi treatment may also be limiting progression, although this has not been a limitation in other cancer types [66,67].

### 2.3. Histone Methyltransferases in OAC

During histone methylation, methyl groups are attached to amino acids of histones. Histone methylation mainly occurs on the side-chain nitrogen atoms of lysines and arginines, most frequently on histones H3 and H4 [68]. Lysine and arginine can both be mono- or di-methylated, whereas only lysine can undergo trimethylation [27]. The methylation process is regulated by histone methyltransferases (HMT) and histone demethylases (HDM). The action of histone methylation can either promote or inhibit gene expression depending on the specific site affected [29]. There are a number of well-characterised lysine methylation marks associated with transcriptional activation including H3K4, which has been shown to be in its tri-methylated state exclusively at active genes [69]. Notable examples of lysine methylation marks associated with transcriptional repression include H3K9 [70] and H3K27 [48]. The latter being methylated by Polycomb Repressive Complex 2 (PRC2), who’s enzymatic activity comes from the SET domain of the core subunit Enhancer of zeste homolog 2 (EZH2) together with the cofactor S-adenosyl-L-methionine (SAM), responsible for healthy embryonic development via epigenetic maintenance of genes with their primary role being in development and differentiation [71].

Whilst there are no reports of EZH2 involvement in OAC tumorigenesis, Frankell et al. (2019) predicted clinically significant sensitivity of OAC cells to EZH2 inhibition [45]. Using a cohort of 551 genomically characterized OACs with matched RNA sequencing data, they identified 77 OAC driver genes and 21 noncoding driver elements [45]. Using the Cancer Biomarkers database, they calculated the percentage of the included OAC cases that contained biomarkers of response to each drug class in the database. Several drugs with predicted sensitivity to >10% of OAC were reported, including EZH2 inhibitors (EZH2i), which are thought to be therapeutic candidates for OAC with SWItch/Sucrose Non-Fermentable (SWI/SNF) complex mutations. The SWI/SNF complex is a subfamily of ATP-dependent chromatin remodelling complexes that modulate transcription and are essential for cell differentiation, proliferation and DNA repair [72]. Mutations in the SWI/SNF complex are observed in 25% of all human cancers and 28% of OAC [73,74]. Functionally antagonistic roles of EZH2 and SWI/SNF have previously been demonstrated, leading to the hypothesis that targeting EZH2 in SWI/SNF mutant cancers could be effective due to synthetic lethality [75]. This has been investigated in ovarian clear cell carcinoma (OCCC) where EZH2i have shown efficacy in OCCC cells with *ARID1A* (the DNA binding component of the SWI/SNF complex) loss of function mutations [76].

### 2.4. Potential for EZH2 Inhibition in OAC

EZH2i have received much of the attention for histone methyltransferase inhibitors and are currently in over ten Phase 1 and 2 clinical trials for treatment of a variety of cancers including B cell lymphoma, metastatic prostate cancer and synovial sarcoma [77]. Six out of the ten EZH2i entered into clinical trials are SAM-competitive inhibitors and act on both the mutant and wild-type forms of EZH2, leading to upregulation of EZH2 target genes. Tazemetostat, an oral SAM-competitive inhibitor, was licensed by the FDA in 2020 for paediatric and adult locally advanced or metastatic epithelioid sarcoma [21], and has also shown promising anti-tumour results in a Phase 2 clinical trial in refractory metastatic mesothelioma [78]. Similarly, a Phase 2 trial in patients with refractory follicular lymphoma has demonstrated efficacy for tazemetostat with an observed treatment response seen in 74% of subjects with an activating EZH2 mutation and a complete response seen in 10% [79], leading to accelerated FDA approval in June 2020 [22]. In contrast, a Phase I trial of patients with haematological malignancies treated with GSK2816126, another SAM-competitive EZH2i, was terminated early due to lack of clinical activity despite anti-tumour effects seen in vitro and in vivo [80,81].

Kofonikolas et al. (2019) investigated the effects of EZH2i (Tazemetostat) on OAC cell lines [37]. Tazemetostat was ineffective in all cell lines, including a SWI/SNF mutant cell line harbouring a *SMARCA4* deletion, despite the predicted OAC sensitivity to EZH2i [45]. However, SMARCA4 is a separate subunit of the SWI/SNF complex than ARID1A, with deletions previously shown to drive EZH2i resistance in OCCC cells [82]. Together with the extremely small sample size (*n* = 1), this may explain why no effect was seen.

## 3. Tumour Microenvironment and HMEs

The heterogenous collection of both infiltrating and host cells that surround tumour cells make up the complex ecosystem of the tumour microenvironment (TME). Whilst the constituents of the TME vary between cancer types, they typically include three broad categories, immune, stromal and cancer cells. Constituents include cancer-associated fibroblasts (CAFs) as the dominant stromal cell type, endothelial cells and extracellular matrix [83,84]. Through complex signalling networks comprising cytokines, growth factors and inflammatory mediators, the TME plays a crucial role in promoting tumorigenesis and drug resistance [85].

### 3.1. Tumour Microenivironment in OAC

Specifically in OAC, a chronic inflammatory state triggered by exposures to bile acid, smoking or alcohol promotes cellular proliferation and apoptotic resistance through cytokines and transcription factors such as interleukin-6, STAT3, Nuclear factor-kappa B and cyclooxygenase 2 [86,87,88]. Given the key role fibroblasts play in chronic inflammation, their presence within the TME is thought to be crucial to tumorigenesis [89]. The pro-tumorigenic phenotype of CAFs is thought to be induced by local secretion of growth factors such as transforming growth factor-β (TGF-β) from the tumour cells [89]. Most OACs contain a myofibroblastic CAF-rich stroma, which through interactions between CAF-secreted periostin and OAC-expressed integrins, cause PI3K-AKT signalling activation and tumour cell invasion [90]. Numerous immune cell types within the OAC TME are involved in promoting an immunosuppressed state that disrupts normal anti-tumour immunity [88]. Regulatory T-cells (Tregs) are seen in increased numbers in both peripheral blood and oesophageal mucosa in OAC and exert their immunosuppressive effects by an inhibition of effector T-cell function [88,91]. Similarly, expansion of the population of immature myeloid cells through pro-inflammatory and tumour-derived molecules such as vascular endothelial growth factor (VEGF) [92] and interleukin-1 [93] leads to Treg induction and subsequent inhibition of effector T-cells [94] and natural killer cells [95].

### 3.2. Impact of HME Therapies on the TME

**Figure 1:**
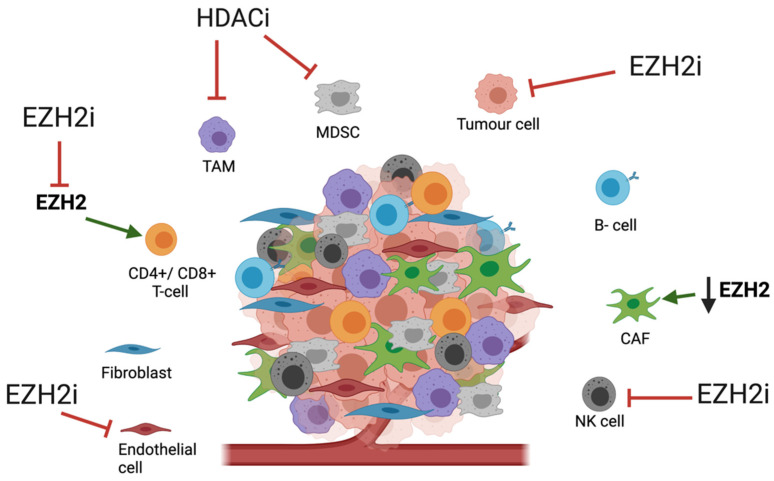
Potential effects of HMEi in the tumour microenvironment. The TME is comprised of numerous cell types which are crucial in promoting tumorigenesis and drug resistance but may be uniquely susceptible to HMEi. EZH2: Enhancer of zeste homolog 2, HDAC: histone deacetylase, TAM: Tumour associated macrophage, CAF: Cancer associated fibroblast, MDSC: myeloid-derived suppressor cells, NK cell: Natural Killer cell. Red arrow: downregulation/inhibition, Green arrow: upregulation. (Created with BioRender.com, accessed on 27 June 2021).

Our increasing knowledge of the role of the TME in cancer has opened many possible therapeutic avenues. The recent focus in TME-targeted therapies in OAC has been on anti-angiogenic agents such as anti-vascular endothelial growth factor agents (anti-VEGF) [96], immune checkpoint inhibitors such as programmed cell death protein 1 (PD-1) inhibitors [97] and therapies to alter the CAF phenotype such as phosphodiesterase type 5 inhibitors (PDE5i) [88]. Whilst the impact of HME-targeted therapies on the OAC TME is unexplored, there are numerous theoretical targets which have been explored in other diseases (Figure 1).

The relationship between EZH2 and activity of TME constituents appears to be tissue-dependent with both pro- and anti-tumorigenic activity. In colorectal cancer mouse models, EZH2 expression was found upregulated in tumour infiltrating Tregs and was shown to be crucial in the Treg-mediated suppression of anti-cancer immunity [98]. Both pharmacological inhibition and gene knockout of EZH2 in these models led to Treg recruitment of CD8+ and CD4+ effector T-cells, resulting in increased anti-tumour activity [98]. Similarly, pharmacological or genetic disruption of EZH2 activity has been shown to enhance natural killer cell maturation and anti-tumour function, which are often repressed in cancer [99]. Tyan et al. (2012) reported that normal tissue-associated fibroblasts that were co-cultured with breast cancer cells gained persistent activity for promoting cancer cell invasion by fibroblast secretion of ADAM metallopeptidase with thrombospondin type 1 motif (ADAMTS1) [100]. This was thought to be due to an observed reduction in EZH2 binding and subsequent loss of the transcriptionally repressive Histone 3 lysine 27 (H3K27) methylation at the promoter region of ADAMTS1 [100].

Small molecule inhibition of HDAC (Trichostatin A) in melanoma, breast cancer and lung cancer mouse models led to a decreased tumour burden and increased overall survival [101]. When examining the role of T-cells in these observations, HDACi was shown to drive trafficking of T-cells to tumours, potentiating the anti-tumour response through CD4+ and CD8+ effector T-cells, whilst decreasing the presence of immunosuppressive immature myeloid cells [101]. When investigating the effects on tumour-associated macrophage (TAM) phenotypes, HDACi abrogated the pro-tumorigenic functions of TAMs in these mouse models [101]. Further to this, several studies have demonstrated that HDAC inhibition can increase the immunogenicity of tumour cells by up-regulating expression of major histocompatibility complex (MHC) proteins and co-stimulatory molecules such as CD40 on the surface of tumour cells, whilst downregulating the expression of anti-apoptotic proteins [102,103,104].

Given the observed role of HDACs in OAC tumorigenesis and the impact of HDACi on the TME in other cancers, targeted HDAC therapy in OAC may be successful in promoting an anti-tumour response within the OAC TME. Similarly, EZH2 inhibition in OAC may potentiate Treg recruitment of effector T-cells and NK cell maturation to increase this anti-tumour response as seen in other cancer types. Due to the impact of immune cell activity within the TME, combining HME therapies with immunotherapies may be key to improving OAC treatment and will be discussed below.

## 4. Combination Therapies

Much of the early success with HDACi has been seen in haematological malignancies, but clinical outcomes in solid tumours remain disappointing. The exact reasons for this observation have not been elicited, but many of the HDACi have short half-lives, which may limit their distribution to solid tumours, necessitating improvements in methods of drug delivery [20]. Sodji et al. (2015) demonstrated improved anti-proliferative effects with targeted HDACi therapy in oral and cervical cancer cells overexpressing folate receptors by conjugating HDACi with folic acid [105]. To further improve the efficacy of HDACi and limit toxicity seen in early clinical trials, combination treatments with chemotherapies or other epigenetic modifiers are being explored.

Combination treatment with HME agents and chemotherapies may be effective due to the impact of chemotherapy on the anti-tumour immune response. In immunocompetent mice implanted with subcutaneous Lewis lung cancer cells, GSK126 (EZH2i) treatment led to accumulation of myeloid-derived suppressor cells (MDSC) and decreased numbers of CD4^+^ T cells and CD8^+^ T cells, which masked the anti-tumour effects of EZH2i. Administration of either gemcitabine or 5-FU led to depletion of these MDSCs, recovering the activity of effector T-cells and potentiating the GSK126 anti-tumour response [106]. The effect of chemotherapies on depleting MDSCs is likely beneficial in OAC given their increased presence with the TME, which may disrupt the activity of an EZH2i.

The synergistic effects of EZH2i with chemotherapy has also been demonstrated in preclinical studies on non-Hodgkin lymphoma (NHL). Tazemetostat in combination with cyclophosphamide, doxorubicin, oncovin, and prednisolone (CHOP) showed significantly greater cytotoxicity than when given separately [107]. Similarly, combining cisplatin and the HDACi panobinostat decreased cell viability and increased apoptosis compared to monotherapy in hypoxia-induced cisplatin-resistant non-small cell lung cancer cells, indicating synergistic anti-proliferative activity [108].

Current standard of care (SOC) chemotherapy in OAC consists of docetaxel, oxaliplatin, leucovorin and 5-FU (FLOT) [109]. With so few patients (20%) demonstrating a clinically meaningful response to this regime, treatment resistance is a major factor in OAC survival rates [12]. In pre-clinical studies, EZH2 is found to be overexpressed in docetaxel-resistant prostate cancers cells [110], cisplatin-resistant breast cancer cells [111] and 5-FU resistant gastric cancer cells [112]. EZH2 knockdown in all three cancer types and small molecule inhibition in the docetaxel-resistant prostate cancer potentiated cancer cell sensitivity to the chemotherapy agents, suggesting that EZH2 inhibition in combination with FLOT chemotherapy could improve treatment sensitivity in OAC.

Aside from chemotherapies, co-administration of HME drugs in combination with novel agents has been investigated with promising early results. EZH2i and epidermal growth factor inhibitors have shown improved anti-tumour effects in colon cancer and gastric cancer cell lines [113,114]. Combination treatment with HMEi and PD-1 inhibitors may be of particular interest in OAC given the recent results of the CheckMate 577 phase 3 clinical trial in patients with oesophageal or gastroesophageal junction cancer. The trial demonstrated improved disease-free survival for patients receiving adjuvant nivolumab (PD-1 inhibitor) compared to placebo [97]. PD-1 receptors are present on activated T-cells and serve as an immunological checkpoint by downmodulating T-cell effector functions when bound to its ligands (PDL-1 or PDL-2) on antigen-presenting cells [115]. OAC tumour cells and tumour-inflitrating lymphocytes have been shown to highly express both PD-1, PD-L1 and PDL-2 [116]. By inhibiting their interaction, nivolumab has been shown to enhance T-cell effector activity and cytokine production [117].

Whilst HMEi and PD-1 inhibitor combination therapy has not been examined in OAC, evidence from other cancer types is encouraging. In prostate cancer, EZH2 inhibition together with PD-1 inhibition significantly enhanced the anti-tumour response with observed intratumoral trafficking of activated CD8+ T-cells and TAMs [118]. The HDACi vorinostat in combination with the PD-1 inhibitor pembrolizumab has demonstrated synergistic anti-tumour activity in metastatic non-small cell lung cancer [119]. Similarly, triple combination of vorinostat, anti-PD-1 and anti-cytotoxic T-lymphocyte-associated protein 4 (CTLA-4) agents in triple-negative breast cancer mouse models resulted in significant increases in anti-tumour activity and overall survival compared to monotherapy [120]. Given the increased presence of Tregs and reduced activity of effector T-cells typically seen in the OAC TME [88,91], these observations of HMEi and PD-1 inhibitor combination therapy highlight a potential avenue for future OAC research.

## 5. Future Considerations

The paucity of information surrounding HMEs in OAC reflects our current knowledge of OAC and underlines why it remains a cancer of unmet need. In order to expand our knowledge of HMEs it is essential we collect further omic data from OAC tumours, which should be compared to paired normal tissues, to gain a better understanding of the genetic and epigenetic pathways underlying the disease process. To further research the role of HMEs in the OAC TME and the impact of HME therapies we desperately need improved 3D cell models that can reproducibly recapitulate the TME seen in patients, mimicking the inner necrotic core and outer layer of proliferating cells within a tumour together with the nutrient, oxygen and growth factor gradients [121]. With HME therapies likely impacting the immune environment within the OAC TME, development of humanised mouse models will be key to evaluating the in vivo impact on immune cells surrounding the tumour.

## 6. Conclusions

Inhibitors of HMEs are already used in clinical practice for a variety of haematological malignancies, with encouraging results seen in numerous ongoing clinical trials. Evidence supports a role of HMEs in oesophageal cancer tumorigenesis; however, less is known about their role and mechanism in the cancer subtype OAC. Early research from in vitro studies on HME therapies in OAC, particularly HDACi, have been promising, with the identification of several novel candidate biomarkers of response. Broadening research to include other HME therapies such as methyltransferase inhibitors may be fruitful. Given observations of poor results seen with HMEi used in isolation in solid tumours, it is likely that the best therapeutic responses will be seen when using them in combination therapies.

Given the significant morbidity and mortality associated with OAC, new treatments are urgently needed. Further research to elicit the underlying epigenetic pathways and characterisation of the TME in OAC is essential to drive the search for epigenetic-based therapies.

## Figures and Tables

**Table 1:** Key findings of pre-clinical studies using HME therapies in oesophageal adenocarcinoma.

Author	Epigenetic Target	Drug	Model	Main Findings
Lohse et al., 2018 [33]	Epigenetic drug panel	163 compounds	OAC cell lines	HDACi displayed significant anti-tumour activity.
Saunders et al., 2017 [34]	HDAC	Pabinostat	OAC 3D-TGA	Pabinostat and standard of care agents improved chemosensitivity of the 3D cell models.
Ahrens et al., 2015 [35]	HDAC DNMT	Vorinostat, MS-275, FK228 AZA, DAC	OAC cell lines	MS-275, FK228 and AZA showed anti-proliferative effects on OAC cell lines.
Feingold et al., 2018 [36]	HDAC	Entinostat	OAC cell lines	Combination treatment with entinostat and cisplatin led to cancer cell apoptosis.
Kofonikolas et al., 2019 * [37]	HDAC EZH2 BET	Vorinostat Tazemetostat JQ1	OAC cell lines	Myc-amplified OAC cells showed increased sensitivity to BET inhibition.

HDAC: histone deacetylase, DNMT: DNA methyltransferase, 3D-TGA: 3 dimensional-tumour growth assay, EZH2: Enhancer of zeste homolog 2, BET: Bromodomain and Extra-Terminal motif protein. * Conference abstract only.

## Abbreviations

3D-TGA	3 dimensional-tumour growth assay
ADAMTS1	ADAM metallopeptidase with thrombospondin type 1 motif
ARID1A	AT-Rich Interaction Domain 1A
AZA	Azacytidine
BET	Bromodomain and Extra- Terminal motif protein
CAF	Cancer-associated fibroblast
CTCL	Primary cutaneous T-cell lymphoma
CTLA-4	Cytotoxic T-lymphocyte-associated protein 4
DAC	Decitabine
DNMT	DNA methyltransferase
DNMTi	DNA methyltransferase inhibitors
ECF	Epiribucin, cisplatin and fluorouracil
EZH2	Enhancer of zeste homolog 2
EZH2i	EZH2 inhibitor
FDA	US Food and Drug Administration
FLOT	Docetaxel, oxaliplatin, leucovorin and 5-FU
H3K27	Histone 3 lysine 27
HAT	Histone acetyltransferase
HDAC	Histone deacetylase
HDACi	Histone deacetylase inhibitor
HDM	Histone demethylases
HME	Histone modifying enzyme
HMEi	HME inhibitors
HMT	Histone methyltransferases
MTA1	Metastasis-associated gene 1
MDSC	Myeloid-derived suppressor cells
NK cell	Natural killer cell
OAC	Oesophageal adenocarcinoma
OCCC	Ovarian clear cell carcinoma
OSCC	Oesophageal squamous cell carcinoma
PD-1	Programmed cell death protein 1
PDE5i	Phosphodiesterase type 5 inhibitors
PRC2	Polycomb Repressive Complex 2
PTCL	Peripheral T-cell lymphoma
qRT-PCR	Real-Time Quantitative Reverse Transcription PCR)
SAM	S-adenosyl-L-methionine
SMARCA4	SWI/SNF Related, Matrix Associated, Actin Dependent Regulator Of Chromatin, Subfamily A, Member 4
SOC	Standard of care
SWI/SNF	SWItch/Sucrose Non-Fermentable complex
TAM	Tumour-associated macrophages
TGF-β	Transforming growth factor-β
TME	Tumour microenvironment
Tregs	Regulatory T-cells
TXNIP	Thioredoxin Interacting Protein
VEGF	Vascular endothelial growth factor
